# Induced Defense in Ryegrass–Epichloë Symbiosis Against *Listronotus bonariensis*: Impact on Peramine Levels and Pest Performance

**DOI:** 10.3390/jof11010050

**Published:** 2025-01-09

**Authors:** Manuel Chacón-Fuentes, Gunnary León-Finalé, Marcelo Lizama, Gastón Gutiérrez-Gamboa, Daniel Martínez-Cisterna, Andrés Quiroz, Leonardo Bardehle

**Affiliations:** 1 Agriaquaculture Nutritional Genomic Center, CGNA, Temuco 4781158, Chile; 2 Programa de Doctorado en Ciencias de Recursos Naturales, Facultad de Ingeniería y Ciencias, Universidad de La Frontera, Temuco 4811230, Chile; gleonfinale@gmail.com (G.L.-F.); d.martinez11@ufromail.cl (D.M.-C.); 3 Programa de Doctorado en Ciencias Agroalimentarias y Medioambiente, Facultad de Ciencias Agropecuarias y Medioambiente, Universidad de La Frontera, Temuco 4811230, Chile; m.lizama04@ufromail.cl; 4 Programa de Doctorado en Ciencias e Ingeniería Agroalimentarias y de Biosistemas, Universidad de Valladolid, 34004 Palencia, Spain; 5 Instituto de Investigaciones Agropecuarias, INIA Carillanca, km 10 Camino Cajón-Vilcún s/n, Casilla 929, Temuco 4880000, Chile; gaston.gutierrez@inia.cl; 6 Escuela de Agronomía, Facultad de Ciencias, Ingeniería y Tecnología, Universidad Mayor, Casilla 54-D, Temuco 4780000, Chile; 7 Laboratorio de Química Ecológica, Departamento de Ciencias Químicas y Recursos Naturales, Universidad de La Frontera, Av. Francisco Salazar 01145, Casilla 54-D, Temuco 4811230, Chile; andres.quiroz@ufrontera.cl; 8 Centro de Investigación Biotecnológica Aplicada al Medio Ambiente (CIBAMA), Universidad de La Frontera, Av. Francisco Salazar 01145, Casilla 54-D, Temuco 4811230, Chile; 9 Departamento de Producción Agropecuaria, Facultad de Ciencias Agropecuarias y Medioambiente, Universidad de La Frontera, Av. Francisco Salazar 01145, Casilla 54-D, Temuco 4811230, Chile

**Keywords:** pest control, insect repellent, induced defense mechanism, peramine, alkaloid, *Listronotus bonariensis*

## Abstract

The Argentine stem weevil (ASW), a major pest in ryegrass pastures, causes significant agricultural losses. Ryegrass can establish a symbiotic association with *Epichloë* endophytic fungi, which supply chemical defenses, including peramine. This symbiosis helps protect ryegrass by providing peramine, which acts as a primary defense. In addition, ryegrass can activate induced defense mechanisms, with peramine remaining the central agent in response to herbivorous insect attacks. Therefore, this study assessed the feeding of the ASW on ryegrass carrying endophytic fungus and peramine levels in aerial organs and its effects on pest performance. Argentine stem weevil adults and larvae were placed on ryegrass leaves and stems to assess feeding. Two treatments were used: endophyte-free plants and endophyte-colonized plants. After ASW feeding damage, insect consumption was measured by the leaf area consumed. To evaluate peramine production and its increase in response to ASW attack, peramine levels in leaves were analyzed using liquid chromatography. Damaged E+ ryegrass plants showed significant increases in peramine, with adult and larval herbivory raising levels by 291% and 216% in stems and by 135% and 85% in leaves, respectively, compared to controls. Endophyte-free (E−) plants experienced more ASW damage, as insects preferred feeding on them, showing reduced activity as peramine levels rose in endophyte-infected (E+) plants. An oviposition assay confirmed insect preference for endophyte-free (E−) plants. Additionally, larvae reared on endophyte-infected (E+) plants had lower survival rates, correlating negatively with peramine levels. These results emphasize peramine’s role in strengthening ryegrass defenses against ASW, impacting both feeding and larval development.

## 1. Introduction

The economic base of livestock production in southern Chile is supported by pastures, the most abundant and cost-effective forage resource [1]. This largely determines the economic viability of animal production systems (milk and/or meat). One of the key factors in beef and dairy production, both in Chile and worldwide, is the correct choice of forage grass and legume cultivars or mixtures to ensure high yields and grassland quality [2,3]. Globally, pasture-based systems play a crucial role in the agricultural economy, providing essential feed for livestock [4]. However, the productivity of these systems is heavily influenced by pest pressures, which can significantly impact forage quality and quantity [5]. As a result, understanding how to sustainably manage pests is a critical challenge in ensuring the long-term viability of pasturelands [6,7]. Perennial ryegrass (*Lolium perenne* L.) is the most popular and economically important grass in temperate regions due to its high nutritional value, digestibility, and forage yield [2,8]. Like all traditional crops, *L. perenne* grasslands are vulnerable to highly destructive herbivorous insects, which can cause significant crop losses. These pests, particularly herbivorous insects, threaten the stability of pasture ecosystems by reducing forage quality and, consequently, animal productivity. In Chile, the Argentine stem weevil (ASW), *Listronotus bonariensis* (Kushel) (Coleoptera: Curculionidae), a native South American species, is a significant pest in ryegrass pastures. *Listronotus bonariensis* larvae bore into the pseudostem (referred to hereafter as stems) and crowns, compromising pasture persistence and productivity, while the adults reduce seedling establishment by defoliating and cutting stems [9,10]. Economic losses due to this pest are estimated at USD 78–251 million per year in New Zealand [11]. In Chile, Cisternas [12] estimated a 44.4% reduction in dry matter production in *L. multiflorum* (annual ryegrass) due to larval attack. The most common method to control this pest is the use of pesticides, which has adverse effects on human and animal health [13,14]. Pesticides are also a primary source of soil contamination [15], are inefficient for borer pest control [16], and often harm the entire invertebrate community due to low specificity [17,18].

Given the growing concern over pesticide use and its environmental and health impacts, researchers are increasingly looking for alternative, eco-friendly pest management strategies [19]. One promising area of research is the use of natural plant–microbe interactions, such as those involving endophytic fungi, which can provide pest resistance without relying on chemical inputs [20]. Endophytic fungi play an essential role in sustainable pest management by interacting with their host plants to enhance resistance against insect pests [21]. These fungi inhabit plant tissues asymptomatically, producing bioactive compounds that can directly deter or inhibit pest activity. Additionally, they can alter plant physiology to create unfavorable conditions for herbivorous insects, such as modifying leaf reflectance or emitting volatile organic compounds (VOCs) that repel pests [22]. Among these fungi, species of the *Epichloë* genus form a particularly significant symbiotic relationship with ryegrass. This symbiosis allows *Epichloë* to inhabit the plant’s epigeal tissues through the apoplast without causing damage, and the fungi are transmitted vertically through seeds, ensuring seedlings are infected upon germination and preventing subsequent fungal acquisition [23,24,25]. The *Epichloë*–ryegrass association offers several advantages to the host plant, including drought tolerance, faster growth, higher seed production, and enhanced defense against herbivory, which can alter insect feeding, performance, and preference [26,27,28,29,30]. This symbiosis produces a “cocktail of alkaloids” with insecticidal, antifeedant, and deterrent effects, where peramine is the primary compound responsible for reducing damage caused by insects such as ASW [31,32,33,34,35]. Such natural defenses significantly contribute to minimizing reliance on chemical pesticides, reducing ecological damage, and decreasing the risk of pest resistance. Furthermore, the potential applications of endophytic fungi in pest control extend to genetically modified strains and synergistic combinations with other microbial agents, offering innovative, environmentally friendly approaches to integrated pest management [22,23].

Plants infected with these endophytes are less susceptible to herbivory by two harmful species, ASW and *Heteronychus arator* (Coleoptera: Scarabaeidae) [35,36]. Moreover, this relationship has broader implications for pest management in grassland ecosystems, as these alkaloids can also impact a range of insect pests beyond the target species. Increased mortality in other insect pests of ryegrass, such as *Spodoptera frugiperda*, *Agrotis ipsilon* (Lepidoptera: Noctuidae), and *Costelytra zealandica* (Coleoptera: Scarabaeidae), has also been observed due to reduced larval weight from decreased food consumption [33,37,38,39]. Some of the alkaloids produced in this symbiosis, such as ergovaline and lolitrem B, are toxic to mammals, causing livestock toxicosis. Eliminating these harmful alkaloids is a major research focus in developing new endophyte strains [40]. In contrast, other alkaloids produced by the symbiont, such as the pyrrolopyrazine peramine, have only an antifeedant and deterrent effect on insects without harming mammals [33,41,42]. The AR1 strain of *E. festucae* var. *lolii* produces high amounts of peramine but neither ergovaline nor lolitrem B. Despite the positive effects of symbiosis, these defenses can be costly for the plant, as they require allocating resources—sometimes limited—for protection rather than growth or reproduction [43], given that the plant provides all necessary nutrients to sustain endophyte survival and alkaloid production. This trade-off in resource allocation raises important questions about how plants balance defensive traits with growth and reproduction under varying levels of pest pressure. To optimize resources, the plant should allocate them to maximize its fitness, protecting tissues based on their importance or potential damage while minimizing the cost–benefit ratio [44,45]. Fuchs et al. [46] observed herbivore-specific induction in the ryegrass–endophyte complex, with the peramine concentration increasing by approximately 60% following herbivory by the locust *Schistocerca gregaria* (Orthoptera: Acrididae) and a 54% increase in lolitrem B following cattle herbivory-like damage, suggesting that the complex can respond efficiently to different types of herbivory. However, the mechanism behind this tissue-specific defense response remains unclear and warrants further investigation. It is possible that the *Lolium–Epichloë* complex also responds variably depending on the damage location (tissue), optimizing resources—a possibility that has yet to be confirmed. Furthermore, the response of other tissues to localized damage and the relationship between response level and damage extent remain unexplored. Additionally, it is unknown how the insect’s performance is affected by the plant’s defense response triggered by its feeding.

Given the open questions in this developing field and considering that ASW is one of the most important pests of *L. perenne*, a study exploring the effect of larval and adult damage on alkaloid concentration across different plant organs would be of theoretical and practical relevance. Consequently, our goals are to (a) assess whether *L. bonariensis* feeding induces a defense response (increase in peramine concentration) in plants infected with endophytic fungus compared to non-infected plants, and whether this response varies based on the plant part consumed and/or damage level.

## 2. Materials and Methods

### 2.1. Plant Material

The *L. perenne* commercial cultivar BASE was used in this study. Two forms of this cultivar were acquired from Universidad de La Frontera (Temuco, Chile); the first corresponds to plants of the BASE cultivar free of endophyte (E−), and the second corresponds to the BASE cultivar infected with the AR1 fungus strain (E+). To ensure the endophyte infection level in endophyte-infected (E+) plants and verify the purity of endophyte-free (E−) plants, a microscopic inspection was conducted following the methodology of Saha et al. [47] and Belanger [48]. Ninety-six percent of the plants were infested with the endophyte. This was confirmed as the standard rose Bengal solution readily stained fungal hyphae in endophyte-infected (E+) plants, whereas in endophyte-free (E−) tissue, only cellular components such as chloroplasts were stained. For plant acquisition from these seeds, 1 kg of seeds was placed on damp filter paper and kept in the laboratory at a constant temperature and humidity (25 °C and 80% R.H.). Five days after germination, the seeds were transplanted into 15 × 15 × 15 cm plastic pots using andisol soil from the La Araucanía Region as the substrate. Two fertilizations with Novatec Classic (NPK) were performed 14 and 40 days after transplant (equivalent to 60 units of nitrogen, 60 units of phosphorus, and 150 units of potassium/fertilization divided into two applications). All fertilizers were purchased from Copeval (Temuco, Chile). The ryegrass was watered every two days and kept under controlled conditions with natural light, a temperature of 25 °C, and humidity of about 80%. All plants were grown for three months.

### 2.2. Listronotus bonariensis Collection and Rearing

Adults used for leaf damage and oviposition assays (see below) were collected in November and December from ryegrass-infested fields in the Los Lagos and La Araucanía regions of Chile. The insects were reared on BASE seedlings under controlled conditions (25 °C and approximately 80% humidity) until used in the experiments. In contrast, neonate ASW larvae for stem damage assays (see below) were obtained from eggs laid by these adults, representing the first generation (F1) of ASW on *L. perenne* (E−) seedlings. Finally, for the collection of each larva from ryegrass plants, no infected leaves were selected, and under a magnifying lens, larvae were visually detected and collected from the damaged leaf using a scalpel and brush. The larvae were then used directly in the experiment.

### 2.3. Damage by Listronotus bonariensis Adults and Larvae

Weevils were randomly assigned to one of two treatments: endophyte-infected (E+) plants and endophyte-free (E−) (control) plants (five adult insects per treatment). This procedure was repeated 20 times for each treatment (*N* = 20). To determine the feeding on the stem by larvae of ASW, plants with two tillers were exposed to ASW larvae. Plants were randomly assigned to one of two treatments: E+ and E− (five newly hatched larvae, born the same day (first larval instar)). This procedure was repeated 20 times for each treatment (*N* = 20). Each stem was observed under a microscope to assess the damage caused by the larvae. In both experiments, plants were kept at 25 °C and watered every two days throughout the duration of the experiment (2 weeks). To assess the damage from adults and larvae, a qualitative scale was used, ranging from 0 to 5, where 0 = no damage; 1 = up to approximately 10% of the leaf surface or stem tissue damaged; 2 = approximately 20–30% damage; 3 = approximately 40–50% damage; 4 = approximately 60–70% damage; and 5 = approximately 80–100% damage [49,50]. To prevent insect escape, each plant was covered with an anti-aphid mesh tent, with the edges sealed using silicone.

### 2.4. Effect of Herbivory on Peramine Production

The peramine concentration in stems and leaves (from Section 2.3) was compared between endophyte-infected (E+) plants damaged by herbivory (adult or larval) and control plants (endophyte-infected (E+) plants without herbivory). Additionally, to evaluate whether the induced response depends on the extent of damage suffered by the plant, a correlation test was conducted between the level of damage in leaves or stems (caused by adult or larval feeding) and the individual induced response (IRi) for each plant. Therefore, to obtain the IRi, the following formula proposed was used:

IRi = PiH − PaC

where

IRi = Individual induced response
PiH = Peramine concentration of the individual plant damaged by *L. bonariensis* adult or larval feeding
PaC = Average peramine concentration in control plants

### 2.5. Peramine Extraction and Analysis by High-Performance Liquid Chromatography

Frozen (−80 °C) ryegrass stems and leaves from adult and larval assays were lyophilized for 24 h using a Biobase lyophilizer (Bioindustry, Jinan, China) to achieve complete desiccation. The dried plant material was then finely milled and precisely weighed using a Radwag AS 220.R2 analytical scale (Radwag, Radom, Poland). Peramine extraction was carried out using a modified method adapted from Roylance et al. [51]. Briefly, 25 mg of the milled plant sample was extracted with 200 µL of methanol and 200 µL of chloroform (Merck KGaA, Darmstadt, Germany), and the mixture was allowed to stand for 30 min to facilitate compound dissolution. Subsequently, 200 µL of hexane and 200 µL of HPLC-grade water (Merck KGaA) were added to form distinct phases. Samples were centrifuged at 14,000× g for 5 min using a Hitachi Koki centrifuge (Hitachi Koki Co., Ltd., Tokyo, Japan), resulting in a three-phase separation: solid residue, an organic phase, and a hydroalcoholic phase. The hydroalcoholic supernatant was carefully collected and filtered through a 0.22 µm hydrophilic PVDF membrane (Merck Millipore, Carrigtwohill, Ireland) to remove any particulate contaminants. Peramine concentration in the filtrate was analyzed using a Shimadzu HPLC system (LC-20A Prominence, Kyoto, Japan) equipped with a C-18 column (250 × 4.6 mm i.d.; 5 µm particle size) maintained at 25 °C. The isocratic mobile phase, modified from Fannin et al. [52] to enhance chromatographic separation, consisted of 90% guanidinium buffer (A) and 10% acetonitrile (B), delivered at a flow rate of 1 mL/min. Peramine identification was performed by comparing the retention times with those of a known standard, as described by Koulman et al. [53]. For quantification, a calibration curve was established using serial dilutions of peramine standard solutions in water, with concentrations ranging from 0.01 to 40 mg/L.

### 2.6. Effect of Endophyte Infection on Insect Feeding

#### 2.6.1. Adult Performance

To test the effect of peramine concentration on the performance of ASW adults, groups of five adults were placed on endophyte-infected (E+) and endophyte-free (E−) plants, with a total of 10 plants per treatment group. The experiment ran for two weeks, after which the number of eggs laid and the extent of plant damage were recorded (see Section 2.3 above for further details). Leaves from each plant were collected separately for peramine concentration analysis. The total number of eggs laid by ASW adults on leaves was then correlated with the peramine concentrations measured in leaves.

#### 2.6.2. Larval Performance

To test the effect of peramine on the larval stage, 20 groups of four newly hatched larvae were fed on endophyte-infected (E+) plants and endophyte-free (E−) plants, respectively. The experiment lasted two weeks, after which the total number of live larvae recovered was recorded. Leaves and stems from each plant were collected for peramine quantification. The total number of live larvae was compared across plants with peramine concentrations [54].

### 2.7. Statistical Analysis

For the comparison of damage categories for both adult and larval ASW, a Chi-square test was used. To count the total number of eggs and larvae, a Chi-square analysis based on the Poisson distribution was used. To compare peramine concentrations between adult- and larval-damaged plants and controls, a two-way ANOVA was performed followed by Tukey test. Correlations between the individual induced responses in leaves and stems, damage levels by ASW adults and larvae, peramine concentration, and egg number were tested using a Spearman correlation test. All analyses were conducted in GraphPad Prism 5, with a significance level of 0.05.

## 3. Results

### 3.1. Damage by Listronotus bonariensis Adults and Larvae

**Figure 1:**
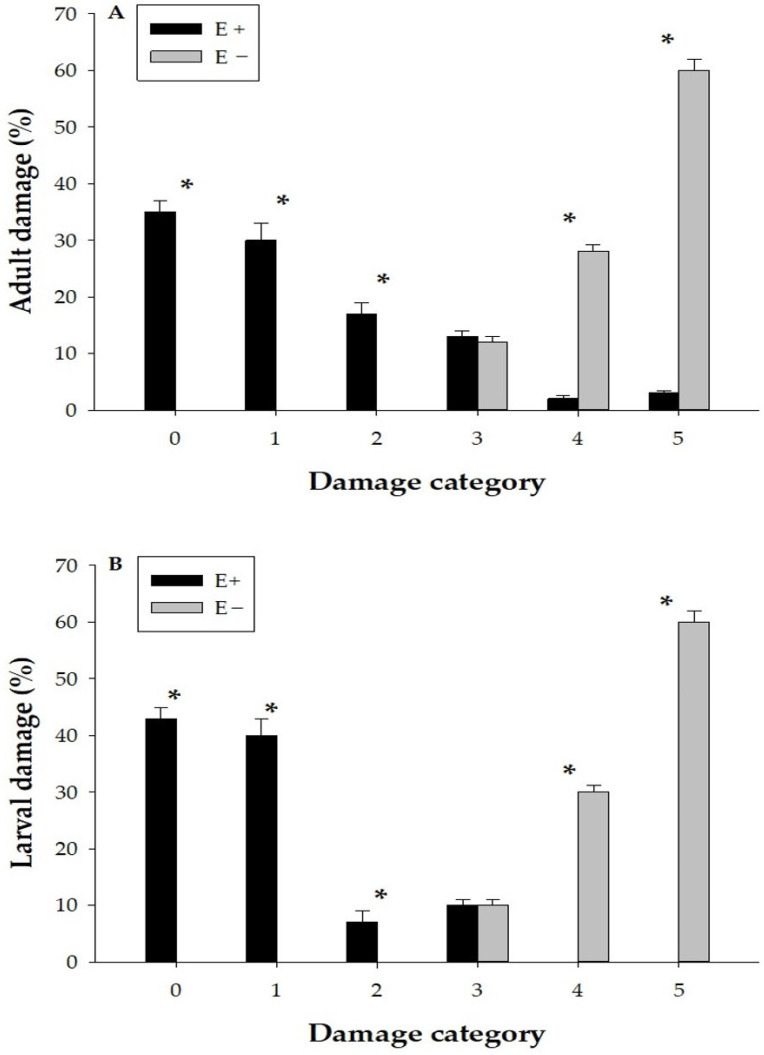
Percentage of leaf damage by *Listronotus bonariensis* adults (**A**) and stem damage by larvae (**B**) on BASE ryegrass infected with AR1 endophyte (E+) and endophyte-free ryegrass (E−) across different damage categories (0–5). Bars represent the mean ± standard error (SE) of damage percentage for each category. Significant differences between E+ and E− within each damage category are indicated by asterisks (*) according to Chi-square test.

Figure 1 shows the percentage of leaf damage by ASW (Figure 1A) and larvae (Figure 1B) on BASE endophyte-infected (E+) ryegrass with AR1 and endophyte-free ryegrass (E−). In both figures, E+ plants generally have lower damage percentages in higher categories (4 and 5) compared to E−. For adult damage (Figure 1A), E+ shows 35% in category 0, decreasing to 3% in category 5, while E− starts at 43% in category 0 and reaches 60% in category 5. For larval damage (Figure 1B), E+ damage remains low in higher categories, showing 0% in categories 4 and 5, whereas E− reaches up to 60% in category 5.

### 3.2. Effect of Herbivory on Peramine Production

**Figure 2:**
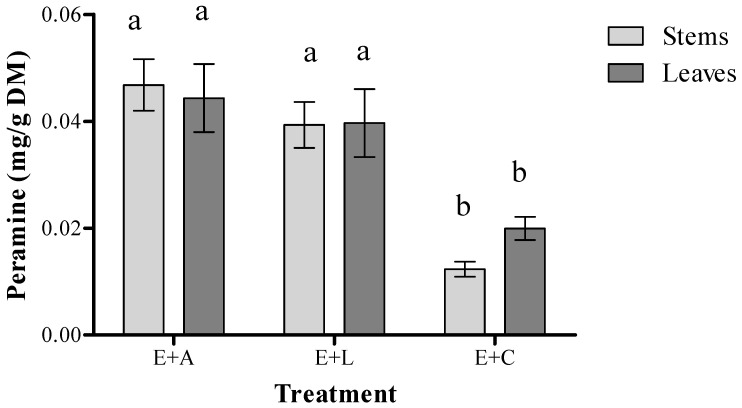
Peramine concentration in damaged and control plants of *Lolium perenne* BASE AR1 cultivar. Damage was produced by both stages of *Listronotus bonariensis*: adults (E + A) and larvae (E + L). Control plants (E + C). Different letters indicate significant differences between treatments (*p* ≤ 0.01) based on a two-way ANOVA followed by Tukey test.

**Figure 3:**
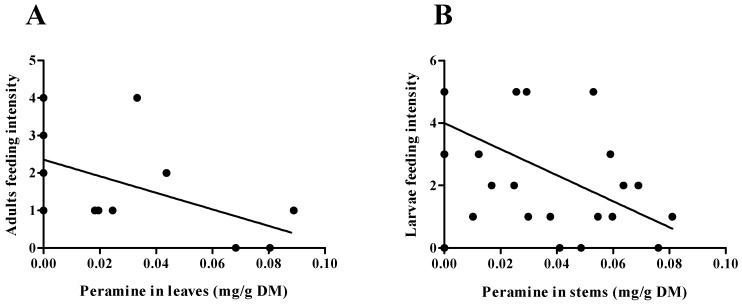
Correlation between peramine concentration in organs of *Lolium perenne* plants and feeding intensity of *Listronotus bonariensis* adults (**A**) and larvae (**B**) herbivory. All the correlations performed were statistically significant (*p* ≤ 0.05) based on a Spearman test.

Significant differences (*p* < 0.001) were found in the peramine concentration in stems and leaves between damaged and control plants (E+), for both adult and larval treatments. Moreover, plants subjected to herbivory showed a higher concentration of peramine (Figure 2). Specifically, the peramine concentration increased by 291% in stems and by 135% in leaves in plants damaged by adult insects compared to control plants. In plants damaged by larvae, the peramine concentration increased by 216% in stems and by 85% in leaves compared to controls. However, no differences were observed in peramine concentration between plant organs within the same herbivory treatment (adult or larval) or between plants with different types of damage (adult vs. larval). Regarding the influence of symbiosis on damage levels caused by ASW, endophyte-free plants (E−) suffered significantly higher levels of damage than endophyte-infected (E+) plants in both adult (t (13) = 1.97; *p* = 0.034) and larval damage assays (t (33) = 5.69; *p* < 0.001). Additionally, there was only a significant correlation between the plant’s induced response (peramine increase compared to control plants, IRi) and the level of damage (R = −0.6367; *p* = 0.014) in the stems of plants damaged by ASW adults. In contrast, no correlation was found between induced response and damage level in leaves damaged by adults or in stems damaged by larvae (*p* > 0.05). When assessing insect performance on plants with varying infestation statuses, both adults and larvae exhibited reduced feeding activity as the peramine concentration increased in leaves and stems (leaves: R = −0.5588; *p* = 0.03; stems: R = −0.6258; *p* < 0.001) (Figure 3). Furthermore, in the feeding preference assay, insects showed a marked preference for endophyte-free (E−) plants as a food source, with significantly higher damage observed on E− leaves compared to E+ leaves (*p* = 0.003). The level of damage on plants was also negatively correlated with the overall peramine concentration (R = −0.5582; *p* = 0.016) (Figure 3).

### 3.3. Effect of Peramine Concentration on Insect Feeding

**Figure 4:**
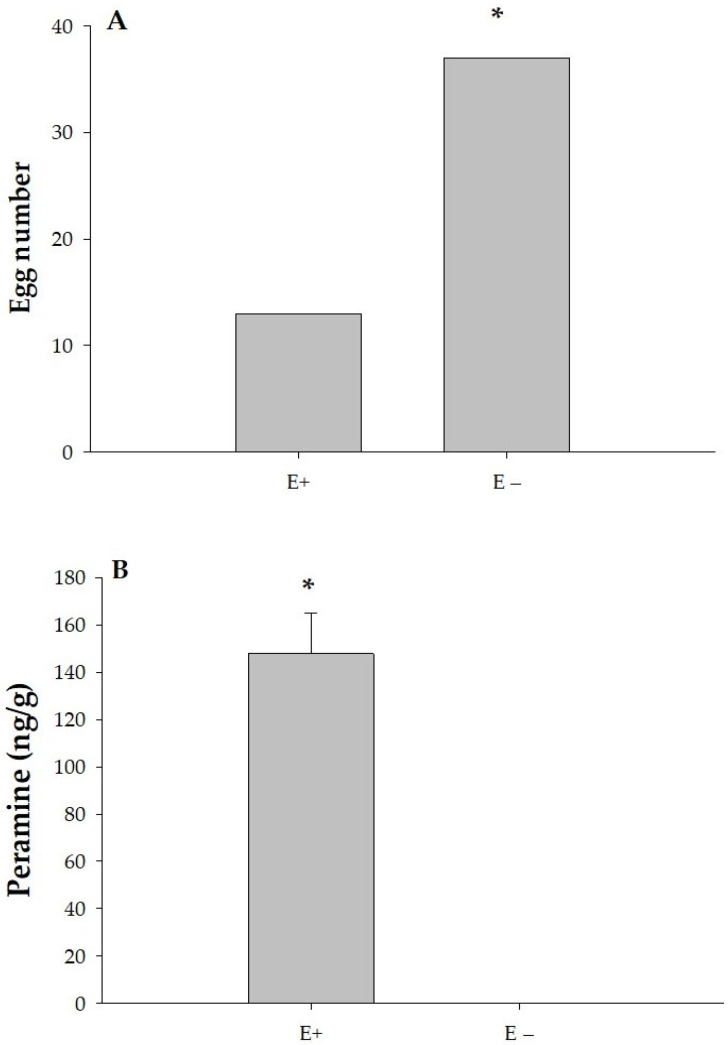
Comparison of the total number of eggs laid by *Listronotus bonariensis* adults on E+ and E− leaves (**A**), and the concentration of peramine in those leaves (**B**). * mean significant difference according to Chi-square test.

In a preference scenario with both endophyte-infected (E+) and endophyte-free (E−) plants available, the total number of eggs was significantly higher on endophyte-free (E−) plants, indicating a preference for these plants as oviposition sites (*p* = 0.04). In Figure 4A, adults laid significantly more eggs on E− leaves (37 eggs) compared to E+ leaves (13 eggs). In Figure 4B, the concentration of peramine, an alkaloid compound, was significantly higher in E+ leaves (147.8 ng/g) compared to E− leaves, which had no detectable peramine (0.0 ng/g). This suggests a correlation between lower peramine levels and higher egg-laying preference by ASW on E− leaves.

**Figure 5:**
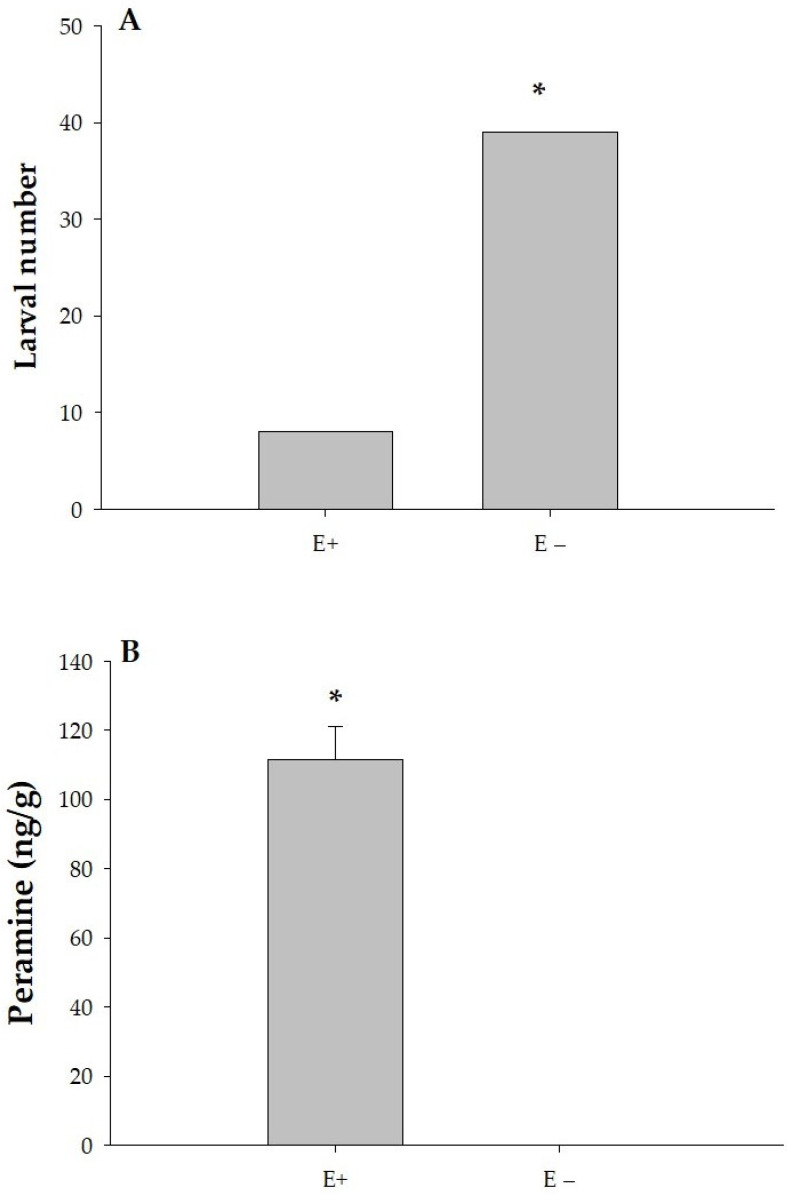
Comparison of the total number of *Listronotus bonariensis* larvae found on E+ and E− leaves (**A**), and the peramine concentration in those leaves (**B**). * mean significant differences according to Chi square test.

In Figure 5A, it is shown that of the total number of larvae found, there were significantly more larvae on E− leaves (39 larvae) compared to E+ leaves (8 larvae). This suggests that E− leaves are more favorable for larval development or survival. In Figure 5B, peramine concentration was significantly higher in E+ leaves, averaging 111.7 ng/g, whereas no peramine was detected in E− leaves (0.0 ng/g).

**Figure 6:**
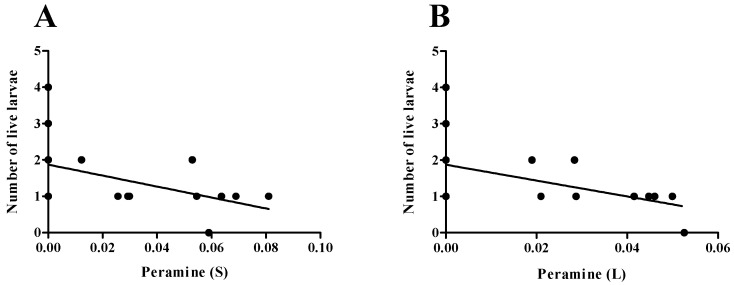
Correlation between peramine concentration in *Lolium perenne* plants and the *L. bonariensis* larvae performance. Number of live larvae related to the peramine concentration in stems (**A**) and leaves (**B**).

Concerning larval fitness, significant differences were observed in the total number of live larvae (*p* = 0.004) between endophyte-infected (E+) and endophyte-free (E−) plants. Larvae reared on E+ plants exhibited poorer performance in both traits compared to those reared on endophyte-free (E−) plants. Additionally, the number of live larvae was negatively correlated with the peramine concentration in stems (R = −0.5461) and leaves (R = −0.4507) (*p* < 0.05) (Figure 6A,B).

## 4. Discussion

The endophytic fungus *E. festucae* var. *lolii* is capable of producing, once associated with the plant, four different alkaloids at different concentrations, with the possibility of finding them simultaneously in the plant [55]. Ergopeptide alkaloids or ergoalkaloids are one of the most commonly found substances in perennial ryegrass, having considerable consequences on the behavior of grazing animals [56]. They have the capacity to produce an increase in the body temperature of the individual with vasoconstriction in the circulatory system, and this generates a cessation in animal feeding, rapid weight loss, and affects its reproductive capacity [57]. The alkaloid lolitrem B is one of the main causes of the so-called “Ryegrass staggers” syndrome, which triggers neuromuscular damage in cattle, sheep, horses and other animals. It causes muscle tremors in the neck and scapula, as well as hyperactivity in coordination, hypermetria, stiff walking, and ataxia in recumbency [58]. Lolines also belong to the alkaloids produced by the endophytic fungus, where despite its presence in *E. festucae* var. *lolii*, it tends to be found more frequently in other species of fungi of the same genus *Epichloë*. This compound has low toxicity in mammals, without causing major health complications, maintaining the corresponding care and in low concentrations. This is not the case for insects, in which direct exposure to the alkaloid can affect them, being toxic to them [59]. Peramine is the fourth alkaloid produced by the fungus *E. festucae* var. *lolii*, which belongs to the substance with the least impact on mammals of those mentioned above. While this is generally true based on current knowledge, peramine is one of the alkaloids produced by *E. festucae var. lolii*, generally considered to have the least impact on mammals compared to the other alkaloids. This alkaloid, unlike the others, is found only in young tissues, being produced in the intercellular spaces where the fungus lodges in the plant. It can be ingested by mammals without representing a danger to their health, but despite this it has a toxic effect on arthropods such as insects. These attributes are beneficial for hosts such as *L. perenne*, which are associated with the endophytic fungus, allowing them to generate a defense against the insect pest ASW [53]. In this sense, peramine could play a role in deterring larval presence on E+ leaves. The results indicate that peramine could contribute to the reduced presence of larvae on E+ leaves, potentially by acting as a deterrent or by influencing the host plant selection of ASW. The elevated peramine concentration observed in the stems and leaves of damaged plants compared to undamaged ones suggests that the plant–endophyte complex can be induced by ASW adults and larval feeding. This aligns with findings by Fuchs et al. [46], who reported a peramine increase from 0.0065 mg/g DM to 0.0104 mg/g DM in *S. gregaria*-damaged plants. In *E. festucae* var. *lolii*-infected *Lolium,* peramine increases seem related to be to herbivory type (chewing insects) rather than insect identity. The higher peramine levels in our study compared to Fuchs et al. [46] may have resulted from the use of an AR1 strain, which only produces peramine, known to yield higher concentrations [52]. This suggests that AR1 endophyte presence reduces damage, especially in severe categories. Moreover, peramine induction appeared to be independent of tissue type and damage intensity. This may be because the endophyte, rather than the plant, produces peramine, potentially responding to plant stress signals or hyphal damage [53]. Factors influencing response intensity could include fungal hyphae quantity or nutrient availability [60,61,62]. While previous studies have evaluated larval or adult herbivory effects separately [25,63], this is the first report comparing adult and larval effects within a single plant–endophyte system. In our study, the peramine increase was systemic (Figure 2), likely due to its high solubility [51,54].

This systemic response could deter ASW, as both adults and larvae were less active in feeding when peramine levels were high. Since ASW has two annual generations [64,65], elevated peramine after adult feeding could preemptively defend the plant against subsequent larval attacks. Peramine’s increase after herbivory may enhance plant defenses, similar to findings in *Festuca pratensis* attacked by *C. zealandica* [63]. The antifeedant effects on ASW adults and larvae align with [50], while similar impacts on larvae were noted in *A. ipsilon* and *Locusta migratoria* (Orthoptera: Acrididae) [25,33]. However, the correlation between peramine concentrations and ASW’s feeding behavior is not absolute, and factors such as environmental conditions or the presence of other compounds could also influence the overall behavior of ASW. This effect may be more critical for larval stages, as they are most damaging to plant development [64] and influence pest survival and reproduction [66,67]. Oviposition behavior only showed a preference for endophyte-free plants (E−) when both endophyte-infected (E+) and endophyte-free (E−) plants were available, suggesting optimal vs. suboptimal resource recognition [68]. Similar selectivity was found by Barker [34], who observed that more eggs were only laid on endophyte-free plants in a dual-choice context. While peramine is not directly toxic to insects, its antifeedant and repellent properties [64,69] lead larvae to starve if they refrain from feeding [25,33,70]. This lack of feeding likely explains the reduced larval size in high-peramine plants, as less feeding results in slower growth, though other factors, such as larval competition, might also influence growth rates [31,71,72]. The presence of endophytic fungi such as the genus *Epichloë* in ryegrass and fescue plants has shown a significant benefit against pests due to the alkaloids produced. However, the development of these endophytes in the plant allows it to provide defense against other harmful agents. There are studies that highlight the effect of the endophytic fungus against the Barley Yellow Dwarf Virus (BYDV), considerably reducing its contagion in endophyte-infected (E+) plants [73], and also against the fungus *Fusarium poae*, where the endophytic fungus *E. festucae* var. *lolii* exhibited a greater production of phenols against the pathogen in relation to endophyte-free plants [74].

In ecological terms, this endophyte-mediated defense mechanism can also influence the population dynamics of ASW by acting as an indirect regulator, limiting both the survival and fecundity of this pest in grass fields. This is significant considering that ASW has two annual generations, which implies that a defense induced by adults can discourage or reduce the presence of larvae in the following generation. Additionally, by selecting endophytes that only produce peramine, farmers can optimize the use of natural defenses in their crops, reducing the need for pesticides and enhancing the sustainability of agricultural practices. This phenomenon, therefore, not only illustrates the sophistication of the defensive response in endophyte-infected plants but also underscores the importance of understanding how these compounds, although not directly lethal, can alter the behavior and host selection in major pests.

## 5. Conclusions

In conclusion, our results show that peramine production in the *Lolium–Epichloë* system can be induced by *Listronotus bonariensis* herbivory, resulting in a systemic increase in this alkaloid in all aboveground tissues of the plant. This increase in peramine concentration occurs regardless of the location or intensity of the damage, suggesting a generalized response of the endophyte to the stress of the host plant. While our findings indicate that peramine acts as a deterrent for *L. bonariensis*, particularly against larvae, it is important to acknowledge that other defensive compounds may also play a role in the plant’s response to herbivory, and further investigation is needed to assess their potential impact on herbivore performance and preference. These findings suggest that peramine likely plays a significant defensive role against *L. bonariensis* attacks, protecting the plant-endophyte system from severe damage and contributing to the indirect regulation of this pest’s population. However, the involvement of other plant compounds in this defense mechanism should be further explored. Ecologically, this induced defense could reduce herbivore pressure on both adult and larval generations of *L. bonariensis*, thus benefiting the longevity and vigor of endophyte-infected grasses. These aspects, however, remain to be studied and evaluated in future research. Therefore, the use of endophytic strains that exclusively produce peramine may represent a sustainable strategy in agricultural systems, but the potential interactions with other defensive compounds should be further considered to fully understand the impact on pest control and overall plant health.

## Data Availability

The original contributions presented in the study are included in the article, further inquiries can be directed to the corresponding authors.
